# Effectiveness of Weekly Supplementation of Iron to Control Anaemia Among Adolescent Girls of Nashik, Maharashtra, India

**Published:** 2008-03

**Authors:** P.R. Deshmukh, B.S. Garg, M.S. Bharambe

**Affiliations:** Dr Sushila Nayar School of Public Health, Mahatma Gandhi Institute of Medical Sciences, Sewagram, Wardha 442 102, India

**Keywords:** Adolescents, Anaemia, Iron-deficiency, Iron, Iron supplementation, Nutrition, Slums, India

## Abstract

A national nutritional anaemia-control programme in India, focusing on supplementation of iron to pregnant women after the first trimester of pregnancy, failed to make an impact. It is prudent to recommend the correction of iron stores before the woman becomes pregnant. ‘Efficacy’ of weekly supplementation of iron has been proved to improve iron stores in adolescence in many studies abroad and in India. The objective was to study the ‘effectiveness’ of a weekly iron-supplementation regimen among urban-slum, rural, and tribal girls of Nashik district, Maharashtra, India. A baseline and the mid-term assessments were done using the cluster-sampling techniques. In each stratum, 30 clusters were identified. Twelve and 10 adolescent girls from each cluster were identified in the baseline and mid-term surveys respectively. The haemoglobin estimation was done using the HemoCue system. Data were analyzed using the Epi Info software (version 6.04). The overall prevalence of anaemia came down significantly to 54.3% from 65.3%. The decline was statistically significant (p<0.001) in tribal girls (48.6% from 68.9%) and among rural girls (51.6% from 62.8%). But the decline was not statistically significant among urban slum girls. Similarly, a significant rise in the mean haemoglobin levels was seen among tribal and rural girls. However, it did not increase significantly among urban slum girls. The programme had performed poorly in urban-slum areas, as the mean number of tablets consumed in urban-slum areas was only 5.6±3.3, as against 6.7±2.6 tablets in tribal girls and 7.2±2.2 tablets in rural girls. Considering the biological and operational feasibility and the effectiveness of the intervention, weekly supplementation of iron to adolescent girls should be universally started to correct the iron stores of a woman before she becomes pregnant.

## INTRODUCTION

Iron-deficiency anaemia is a serious public-health concern in most developing countries. In India, the prevalence of anaemia among adolescent girls is 90% (1). It results in increased maternal mortality and decreased child survival, as supplementation during pregnancy fails to restore the iron status (2). Iron-deficiency anaemia is estimated to cause 591,000 perinatal deaths and 115,000 maternal deaths globally (3). The associated loss of healthy life-years amounts to more than 19 million disability-adjusted life-years (DALYs) from perinatal causes and more than three million from maternal causes (3). When direct sequelae of iron-deficiency anaemia are added, the global burden attributed to iron-deficiency anaemia amounts to 841,000 deaths and 35,057,000 DALYs (3). An effective supplementation programme will prevent part of this loss. Cost-benefit analysis of iron-fortification and supplementation programmes showed that the cost-benefit ratio exceeds unity by a considerable margin, and therefore, it represents a highly productive investment for developing countries (4). In 1993, the World Health Organization (WHO) also recommended actions for the development of assessment, advocacy, prevention, and control initiatives, in most countries, to reduce anaemia among adolescent girls.

‘Efficacy’ of weekly supplementation of iron has demonstrated an improvement in iron stores in adolescence in many studies in India (5-7) and abroad (8-10). The objective of the present study was to study the ‘effectiveness’ of a weekly iron-supplementation regimen through the government health system among urban-slum, rural, and tribal girls of Nashik district.

## MATERIALS AND METHODS

The study was carried out in Nashik district of Maharashtra state, India. The United Nations Children's Fund, in collaboration with the Public Health Department, the Tribal Welfare Department, the Education Department, and the Women and Child Development Department, initiated the ‘Adolescent Nutritional Anaemia Project’ for unmarried, non-pregnant, adolescent girls, aged 14–18 years, in Nashik district from 2000 onwards. The Project covered 498,793 people from four tribal, four rural blocks of Nashik district, and all the urban slums of Nashik city. These girls were given weekly supplementation with iron folic acid tablets (100-mg iron and 0.5-mg folic acid) and were trained in life-skill training sessions for three hours every day for three days. The training module aimed at educating the beneficiaries on nutrition and adolescent issues, such as family and gender differences, adolescence and accompanying changes, and pregnancy and related issues. This opportunity was also used for explaining the importance of consumption of iron folic acid tablets and ensuring compliance over the period. Anganwadi Workers of the Integrated Child Development Services Scheme carried out these activities in their areas. The distribution of iron folic acid tablets was facility-based, i.e. anganwadi. The supplies were replenished during their monthly review meetings at the Primary Health Centre.

The baseline assessment was done during 31 March–April 2001 using the cluster-sampling techniques. Considering the 50% prevalence of anaemia, the sample size required at 7.5% precision, alpha of 5% with design effect of 2, was 342. In each stratum (tribal, rural, and urban-slum), 30 clusters were identified. In total, 12 adolescent girls from each cluster were identified following the standard procedure and were included in the study, after obtaining verbal consent (11). A predesigned and pretested questionnaire was used for collecting data. The HemoCue system was used for estimating haemoglobin (12). Anaemia was defined when the haemoglobin levels were less than 120 g/L. The haemoglobin levels between 100 and 119 g/L were classified as mild anaemia, 70 and 99 g/L as moderate anaemia, and less than 70 g/L as severe anaemia (2).

The same methodology was used for evaluating the programme after 30 months of the intervention from 28 November to 12 December 2003. Considering the 50% prevalence of anaemia, the sample size required at 7.5% precision, alpha of 5% with design effect of 1.8 (calculated from the baseline study using the Epi Info software (version 6.04), was 308. From each cluster, 10 adolescent girls were identified, instead of 12, in the baseline assessment. The decision was also influenced by the fact that, during the baseline assessment, it was very difficult to get 12 adolescent girls from each cluster, as most adolescent girls in rural and tribal areas go out to work, and it, therefore, proved operationally difficult. A two-month recall period was used for evaluating the compliance. During these two months, an adolescent girl was expected to consume a maximum of nine tablets.

### Statistical analysis

Data, thus, collected were analyzed using the Epi Info software (version 6.04). The CSample program of the Epi Info software was used for finding out the variance as data were collected by the cluster-sampling technique. Two tailed *t*-test was used for comparing the means. The chi-square trend was used for studying the trend in different grades of anaemia.

## RESULTS

At baseline, the overall prevalence of anaemia was found to be 65.3%. It was the highest among the tribal girls (68.9%), followed by 64.2% among urban-slum girls, and least (62.8%) among rural girls (Table 1). The overall mean haemoglobin level was 110.7±18.3 g/L. The mean haemoglobin levels among tribal, rural, and urban slum girls were 108.2±19.5 g/L, 112.6±17.0 g/L, and 111.2±18.2 g/L respectively (Table 2).

**Table 1:** Baseline and mid-term prevalence of anaemia

Area	Baseline prevalence (n=360)	Mid-term prevalence (n=300)	p value
Tribal	248 (68.9)	146 (48.6)	<0.001
Rural	226 (62.8)	155 (51.6)	<0.001
Slum	231 (64.2)	188 (62.7)	>0.05
Total	705/1,080 (65.3)	489/900 (54.3)	<0.001

Figures in parentheses indicate percentages

**Table 2:** Mean haemoglobin levels at baseline and mid-term

Area	Baseline haemoglobin (mean±SD) (n=360)	Mid-term haemoglobin (mean±SD) (n=300)	p value
Tribal	108.2±19.5	116.2±15.4	<0.001
Rural	112.6±17.0	115.6±16.3	<0.05
Slum	111.2±18.2	110.3±20.6	>0.05
Total	110.7±18.3	113.7±17.7	<0.05

The overall prevalence of anaemia came down significantly at the time of evaluation to 54.3% from 65.3%. The decline was statistically significant (p<0.001) among the tribal girls (48.6% from 68.9%) and among the rural girls (51.6% from 62.8%). However, the decline was not statistically significant among the urban slum girls (Table 1). Similarly, a significant rise in the mean haemoglobin levels was observed among the tribal and rural girls. The overall mean haemoglobin level also increased significantly (p<0.05) from 110.7±18.3 g/L to 113.7±17.7 g/L (Table 2). A significant declining trend was observed in different grades of anaemia among the tribal and rural girls. The trend was not statistically significant among the urban slum girls (Table 3).

**Table 3:** Grades of anaemia at baseline and mid-term

Area	Baseline (n=360)	Mid-term (n=300)	p value (χ^2^-trend)
Tribal			
No anaemia	112 (31.1)	151 (50.8)	
Mild anaemia	147 (40.8)	106 (35.7)	
Moderate anaemia	88 (24.4)	40 (13.5)	
Severe anaemia	13 (3.6)	0	<0.001
Rural			
No anaemia	134 (37.3)	141 (47.6)	
Mild anaemia	165 (46.0)	110 (37.2)	
Moderate anaemia	53 (14.8)	44 (14.9)	
Severe anaemia	8 (1.9)	1 (0.3)	<0.001
Slum			
No anaemia	129 (35.9)	107 (36.3)	
Mild anaemia	143 (39.8)	119 (40.3)	
Moderate anaemia	81 (22.6)	57 (19.3)	
Severe anaemia	7 (1.7)	12 (4.1)	>0.05

Figures in parentheses indicate percentages

The programme performance in the urban-slum area was not satisfactory as a lesser number (78.7%) of girls had attended the session of Life Skill Education. Only 82.7% of the girls consumed iron folic acid tablets during the last two months against 92.0% by the tribal girls and 94.7% by the rural girls. Similarly, the mean number of tablets consumed during the last two months in the urban-slum area was 5.6±3.3 only as against 6.7±2.6 tablets by the tribal girls and 7.2±2.2 tablets by the rural girls (Table 4).

**Table 4:** Programme performance in tribal, rural, and slum areas

Area	No. attended in life-skill training (n=300)	Consumption of IFA during the last two months	
		No. consumed (n=300)	Consumption (mean±SD) (n=300)
Tribal	236 (78.7)	276 (92.0)	6.77±2.6
Rural	243 (81.0)	284 (94.7)	7.15±2.2
Slum	236 (78.7)	248 (82.7)	5.63±3.3

Figures in parentheses indicate percentages; IFA=Iron folic acid

## DISCUSSION

Nutritional anaemia is an end-result of long-term negative iron balance, culminating in poor immune function and decreased work performance. Poor foetal outcome may occur if iron deficiency occurs in the first trimester of pregnancy (13). The national nutritional anaemia-control programme in India, which focused on supplementation of iron to pregnant women after the first trimester of pregnancy (14), failed to make an impact—similar to the experience from other countries (15). Over the past decade, the proportion of low-birthweight babies has failed to come down (16). This is directly related to child survival and to the physical and mental growth of children as nutritional anaemia in women increases the risk of maternal mortality, reduces the gestational period, increases the risk of low-birthweight, and increases perinatal mortality (17).

Prevention of iron deficiency during pregnancy and young childhood is essential as it can cause the most lasting damage (18). As interventions during pregnancy have failed, it is necessary to correct the iron status even before the woman becomes pregnant. Because of considerable overlap of the increase in iron requirements due to growth, onset of menses, and costs of pregnancy, there is a limited opportunity to acquire a sufficient iron store before pregnancy to meet the demands of pregnancy (19). This makes iron supplementation during adolescence very essential. This will assure that mothers enter pregnancy with adequate iron status.

Supplementation of iron has been recommended in adolescent years (20) mainly because of three factors: first, 16–55% of girls are already anaemic by the time they become pregnant; second, pregnancy is too short a period of time in which to reduce pre-existing anaemia; third, intervention channels already exist which can be used for targeting adolescent girls with iron intake (21,22).

The effectiveness of daily iron-supplementation programmes has been questioned because of the low efficiency of health services and the lack of compliance of the target groups (6). The use of intermittent supplementation schedules has been suggested as a way to improve compliance by reducing side-effects. Several studies conducted among preschool and school-age anaemic children have shown that the efficacy of an intermittent iron-supplementation schedule is similar to that of daily dosing (6,7).

The present study has shown the effectiveness of the weekly supplementation of iron folic acid tablets in rural and tribal areas where the programme performed better. In urban slums, the programme needs to be improved. Alternative modalities, such as peer educators, school-based approach, or house-to-house approach to improve the compliance may be worked out. Implementation of the programme through existing resources makes the intervention sustainable. Mehta also reported an increase in the mean haemoglobin level from 10.445+1.21 g/dL to 11.99+1.19 g/dL and the halving of the prevalence of anaemia from 63.8% after weekly supplementations of iron and folic acid for 25 weeks (5), which were comparable with daily supplementation. Sharma *et al*. also reported that the public-health approach, consisting of a once weekly iron supplementation through schools and welfare centres, may turn out to be a better strategy to combat anaemia in adolescent girls (23). A meta-analysis of intermittent supplementation of iron concluded that weekly supplementation of iron is also efficacious though less than the daily regimen and recommended that, supervision of iron supplementation whether daily or weekly, is essential and situations where daily supervision of iron supplementation is not possible, weekly supervised supplementation may be the feasible alternative (24).

To conclude, considering the biological and operational feasibility and the effectiveness of the intervention, weekly supplementation of iron to adolescent girls should be universally started in rural and tribal areas to correct the iron stores of women prior to becoming pregnant.

## ACKNOWLEDGEMENTS

The authors acknowledge the financial assistance provided by UNICEF. The authors also express their gratitude towards the District Health System, District Women and Child Development Department, and adolescent girls of Nashik.
